# The Mucosal Adjuvant Potential of Cross-Linked Dextran Microspheres as Dry Powder

**Published:** 2012

**Authors:** Mohsen Tafaghodi, Maryam Eskandari

**Affiliations:** 1 *School of Pharmacy, Mashhad University of Medical Sciences, Mashhad, Iran*; 2 *Nanotechnology Research Center, Mashhad University of Medical Sciences, Mashhad, Iran*

**Keywords:** Cross-linked dextran microspheres, Dry powder, Nasal immunization, Quillaja saponins, Tetanus toxoid

## Abstract

**Objective(s):**

The immunoadjuvant potential of cross-linked dextran microspheres (CDM) as absorption enhancer and Quillaja saponins (QS) as immunomodulator adjuvant was evaluated.

**Materials and Methods:**

CDM loaded or tetanus-mixed toxoid (TT) or Quillaja saponin (QS) were nasally administered to rabbits in dry powder form, three times in 2 weeks interval and serum IgG and nasal lavage sIgA titers were determined by ELISA.

**Results:**

The highest serum IgG titer was induced by parenteral immunization through alum adsorbed TT (*P*< 0.001). Among nasally immunized groups, the highest serum IgG titer was induced by (TT+QS)_CDM_ (*P*< 0.01). Mixing of CDM with TT+QS powders (CDM+TT+QS), could not induce the high serum IgG titers as (TT+QS)_CDM_ (*P*< 0.01). CDM loaded with TT+QS induced higher IgG titers than CDM loaded with TT alone (*P*< 0.01). No significant difference was observed in nasal lavage sIgA titers of various groups.

**Conclusion:**

CDM microspheres loaded with TT+QS significantly increased serum anti-TT IgG titers, but mixing of CDM with TT+QS powder could not increase IgG titers. Both QS and CDM adjuvant could not significantly increase the lavage anti-TT IgA titers.

## Introduction

Most of the present vaccines are administered parenteraly and encountered with several disadvantages. Mucosal delivery of vaccines could be a highly effective route for induction of local and systemic immunity  (1). Among the mucosal routes, nasal immunization has good potentials for induction of systemic and mucosal immune responses (1, 2). Because of low permeability of mucosal epithelia to most antigens, using efficient adjuvants is needed. Recently, various delivery systems and adjuvants, like trimethylchitosan (TMC) nanoparticles  (4), oligomannose-coated liposomes (3), nanoemulsions (4), chitosan-coated liposomes (5), a modified pulmonary surfactant named Surfacten (6) and a mutant TNF- alfa (7) have been used for mucosal immunization against Influenza, TB and other infections. 

Among the immunoadjuvants, absorption enhancer adjuvants such as cross-linked dextran microspheres (CDM, Sephadex^®^) could be utilized to overcome the mucosal barriers and increase immune responses  (10). Bioadhesive polymers such as dextran could decrease the mucociliary transport rate and prolong the residence time in the nasal cavity, by the help of which contact time of antigens with specialized antigen sampling cells (M cells) and resulting immune responses will be increased (8, 9). CDM should be administered in dry powder form to be able to absorb water and exert its penetration enhancer effect. 

Formulation of vaccines in dry powder form has several advantages. More chemical and microbiological stabilities of vaccines in dry form could eliminate the need for cold chain. As a result, storage, distribution and mass vaccination is easier and more economical than liquid-based vaccines (10, 11). Therefore antigens are better to be used in dry powder form.

Among the natural immunoadjuvants, the adjuvant effect of *Quillaja* saponins (QS) has been shown in several studies. The purified *Quillaja* saponins (Quil A) are now approved for veterinary vaccines and these are commercially available vaccines, like bovine respiratory syncytial virus vaccine, adjuvanted with QS (12, 13). A purified fraction of QS, named QS21, is also frequently used in clinical trials (12, 14). 

At the present study, CDM powder as an absorption enhancer adjuvant and *Quillaja* saponins (QS) as immunomodulator adjuvant were used for nasal immunization against a model antigen, tetanus toxoid. Powder formulations adjuvanted with CDM, QS or both were nasally administered in rabbits and their impacts on mucosal and systemic immune responses were evaluated. 

## Materials and Methods

***Materials***

Purified *Quillaja *saponin was purchased from Sigma (St Louis, USA). Cross-linked dextran microsphere (Sephadex^®^ G-150) was from BioGene (Sweden). Tetanus toxoid (TT) 2700 Lf/ml and alum-adsorbed TT 50 Lf/ml were obtained from Razi Institute. (Hesarak, Iran). Each Lf of TT was equivalent to 5 µg protein, as determined by BCA protein assay. Anti-rabbit IgG and IgA antibodies were from Sigma (Missouri, USA) and Bethyl Laboratories Inc. (Texas, USA).

White albino rabbits weighing 2–2.5 kg were provided by Pasteur Institute (Tehran, Iran).

***Loading of CDM with TT and/or QS***

Ten mg of CDM powder was added to 40 Lf TT and/or 20 µg QS solution and were mixed in ambient temperature for 10 min. The mixture was freeze-dried and the resulting powder was used for nasal immunization. In some of experimental groups, TT or TT+QS solution were first freeze-dried and resulting powder was mixed with CDM. 

***Morphology and particle size determination***

**Figure 1:**
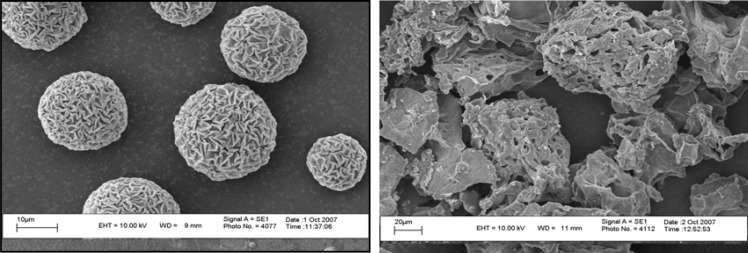
Scanning electron micrograph of CDM microspheres before (A) and after (B) freeze drying

Optical microscope (Olympus, Japan) was used for studying morphological features of CDM before and after freeze-drying. Scanning electron microscope (Leo, Germany) was used for studying the morphology and size of microspheres (Figure 1). For sample preparation, one drop of concentrated suspension of microspheres was dropped on the stub and left in ambient temperature to be dried. Microspheres were coated with gold-palladium in sputter-coater.

***Nasal immunization studies***

White albino rabbits weighing 1.5–2.5 kg (four animals per group) were nasally immunized with the following formulations in days 0, 14 and 28 of experiment. (1Two hundred µl of TT-Sol (100 µl in each nostril) or 10 mg of powder (all groups except for TT-Sol and alum-TT groups, 5 mg in each nostril) were nasally administered. For nasal administration of dry powder of microspheres, powders were filled in polyethylene tubes (2 mm in diameter) and connected to a syringe. Tubes were inserted in animal nose (0.5 cm) and 10 ml of air was blown in tube. Each animal was bled in 3rd and 6th weeks. In the 6th week, the nasal cavity was washed with 5 ml sterile PBS buffer for determination of mucosal sIgA titer.

**Figure 2:**
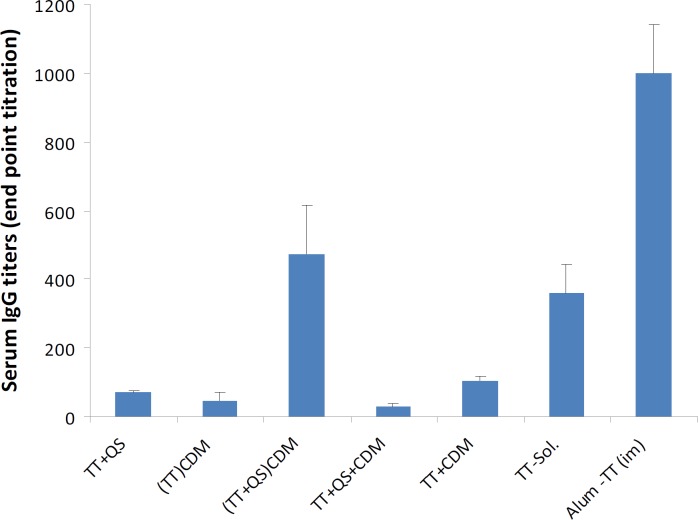
Serum anti-TT IgG titers (mean±SD).

Rabbits (n= 4) were nasally (intramuscularly for Alum-TT) immunized with 40 Lf TT and 20 µg QS, at weeks 0, 2 and 4 and were bled at weeks 3 and 6. Sera anti-TT IgG titers (end point titration) were determined by an ELISA method

***Determination of serum anti-TT IgG titers and nasal lavage anti -TT IgA titers***

Anti-TT antibodies in the rabbit serum and nasal lavage were detected and quantified by end-point titration using an ELISA assay (15). 

End-point titers were determined as the highest dilutions with absorbances equivalent to the normal sera.

***Statistical analysis***

Statistical analysis was carried out by one-way ANOVA and unpaired student’s t test. *P*-values less than 0.05 were regarded as significant.

***Ethics in animal investigations***

The protocols of animal studies were approved by Regional Ethics Committee.

**Figure 3:**
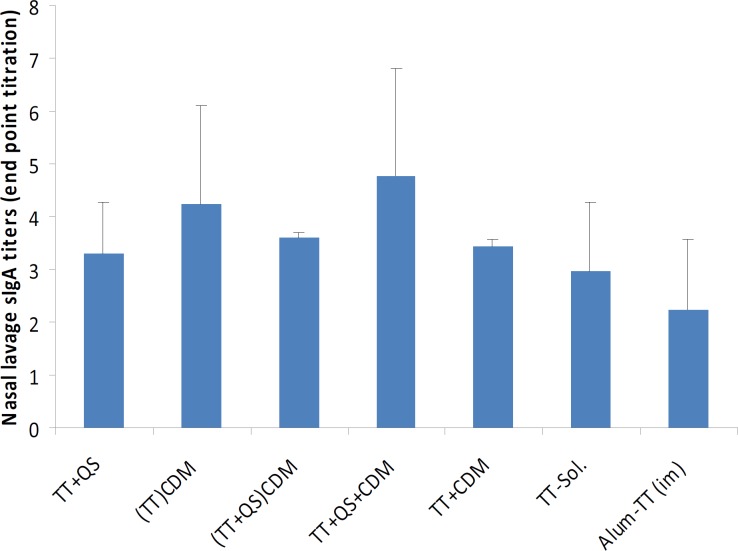
Nasal lavage anti-TT sIgA titers. Rabbits

(n= 4) were nasally (intramuscularly for Alum-TT) immunized with 40 Lf TT and 20µg QS, at weeks 0, 2 and 4 and nasal lavages were collected at week 6. Lavages were pooled and Anti-TT IgA titers (absorbance) were determined by an ELISA method

## Results

***Morphology and size of CDM microspheres***

The spherical and smooth CDM microspheres were converted to particles with irregular shapes after freeze-drying process (Figure 1). Mean diameter of CDM microspheres was determined by direct measurement of diameters of 100 microspheres under optical microscope, equipped with an eyepiece reticule. The mean diameter was 17.4±9.4 µm.

***Serum anti-TT IgG titers***

Rabbits (n= 4) were nasally immunized with 40 Lf TT and 20 µg QS at weeks 0, 2 and 4 and were bled at weeks 3 and 6. Sera anti-TT IgG titers were determined by an ELISA method (Figure 2). Among nasally immunized groups, the highest IgG titer was related to CDM microspheres loaded with TT and QS (*P*< 0.01). When freeze-dried powder of TT was mixed with CDM microspheres, the IgG titer was higher than groups immunized with TT-loaded CDM microspheres (*P*< 0.01) and TT solution (*P*< 0.05). There was no significant difference between IgG titers induced with nasal administration of TT solution and freeze-dried powder of TT+QS and TT-loaded CDM microspheres (*P*> 0.05). Positive control group were intramuscularly injected with 10 Lf Alum-TT and showed the highest IgG titers (*P*< 0.001). 

***Nasal lavage anti-TT IgA titers***

Following the above-mentioned immunizations, at the 6^th^ week nasal lavage was collected, pooled and anti- TT IgA titers were determined by an ELISA method (16). No significant difference was observed among the groups immunized with various formulations (Figure 3) (*P*> 0.05). 

## Discussion

In mucosal immunization, the microfold (M) cells were thought to be the principal uptake site of particulate antigens. These cells which are found in the distal regions of the nose, the nasopharyngeal and palatine tonsils and bronchial associated lymphoid tissues (BALT) in the lung could absorb particles of 1-5 µm in diameter (17, 18). The CDM microspheres with a mean diameter of 17.4±9.4 µm could not be absorbed by M-cells or phagocyted by APCs. Therefore, the porous CDM microspheres (Figure 1) could only have an indirect effect on the responses. 

The need to cold chain for storage and distribution is a restricting factor for massive vaccination, especially in developing countries. One strategy for resolving the problem is preparation of stable powder vaccines. Dry powder formulations are more stable against chemical and microbial destabilization and could eliminate the necessity of the cold chain (10).

At the present study CDM microspheres, commercially available as Sephadex^®^, were examined as absorption enhancer and excipient for nasal formulations. CDM powder was loaded or mixed with TT as a model antigen and QS as an adjuvant its impact on immune responses was evaluated. 

The results presented herein indicate that *Quillaja* saponin (QS) as an adjuvant and cross-linked dextran microspheres (CDM) as an absorption enhancer adjuvant could positively affect the systemic IgG titers. Intranasal administration of (TT+QS)_CDM_ microspheres in powder form showed the highest serum IgG titers (*P< *0.001). The end point titer (EPT) induced with this formulation was half of EPT induced with IM injection of alum-adsorbed TT (*P< *0.001), which is indicative of a potent mucosal adjuvant system. When TT powder was mixed with CDM, induced higher IgG titers than TT+QS powder (*P< *0.05), therefore the adjuvant potential of CDM is higher than QS. 

The IgG titers induced with (TT)_CDM_ was similar to CDM mixed with TT powder (*P*> 0.05). However, (TT+QS)_CDM_ induced significantly higher IgG titers than CDM+TT+QS (*P<*0.001). This implies that loading of CDM microspheres with TT+QS could highly increase its adjuvant potential. Based on previous studies on CDM microspheres for nasal delivery of peptide and protein drugs, absorption of water by the microspheres from the mucus layer may Induce reversible shrinking of the epithelial cells and widening of the tight junctions for about 15 min. In this time, the transport of hydrophilic compounds could be increased (19, 20). Additionally, the absorbed water will dissolve the loaded drug and high concentrations of drug will be formed in contact with epithelial surface (21). It seems that the higher IgG titers induced with (TT+QS) _CDM _could be attributed to the simultaneous delivery of antigen and adjuvant, while delivery of TT alone has not a significant impact. 

**Table 1:** Details of formulations used for *in vivo* immunizations

Formulation	TT (Lf)	Dosage form	CDM (mg)	QS (µg)	Inert powder (lactose) (mg)	Administration route
TT mixed with QS TT+QS	40	Dry powder	-	20	10	i.n.
CDM loaded with TT (TT)_CDM_	40	Dry powder	10	-	-	i.n.
CDM loaded with TT+QS (TT+QS)_CDM_	40	Dry powder	10	20	-	i.n.
CDM mixed with TT+QS TT+QS+CDM	40	Dry powder	10	20	-	i.n.
CDM mixed with TT TT+CDM	40	Dry powder	10	-	-	i.n.
TT solution TT-Sol	40	Solution	-	-	-	i.n.
Alum-adsorbed TT Alum-TT	10	Suspension	-	-	-	i.m.

In several studies the porous cross-linked dextran and starch microspheres have been used for mucosal delivery of insulin (22-25), HCG (26), octreotide (27), G-CSF (28). But there are few reports about using of these microspheres for mucosal delivery of antigens (29). There are controversial results reported in the literature after mucosal immunization by these microspheres (29-31). However, in recent oral immunization studies, cross-linked dextran and starch microspheres smaller than 5 µm in diameters showed better results (32-35). 

In our previous nasal immunization study in rabbits, the immunoadjuvant potential of CDM loaded with TT was compared with TT and TT+ CpG ODN solutions. Among the nasally immunized animals, the highest antitoxin titers was seen in group immunized with CDM+TT (*P*< 0.0001). The serum IgG titers of the CDM+TT group was higher than the TT solution group (*P*< 0.05). The adjuvant potentials of CDM and CpG-ODN in inducing 

IgG titers was not significantly different (*P*> 0.05) (21). 

The nasal sIgA titers induced with different formulations was also studied. Among the groups studied the highest sIgA titers were seen in groups immunized with CDM-containing formulations (Figure 4). The highest titer was seen in TT+QS+CDM and followed by (TT) _CDM_ group. The lowest titers were seen in groups immunized with i.m. injection of Alum-TT and i.n. administration of TT sol. All differences seen in sIgA titers were not statistically significant. In our previous studies, PLGA nanospheres and alginate microspheres encapsulated with TT were mixed with CDM and administered as dry powder. CDM could increase the sIgA titers compared with lactose powders (11, 36). In another study, the nasal sIgA titers induced by CDM+TT was lower than TT solution (21). It seems that mixing of particulate delivery systems like nanospheres and microspheres with CDM can increase the systemic immune responses, but its effect on mucosal antibodies is marginal. 

In nasal delivery of vaccines, the residence time of antigen on the nasal mucosa is very important (37). Mucoadhesive compounds have been used to prolong the residence time on a mucosal surface (10). CDM microspheres were reported, in previous studies, to have high mucoadhesion potential (38). 

## Conclusion

After nasal immunization, CDM microspheres loaded with TT+QS significantly increased serum anti-TT IgG titers, but mixing of CDM with TT+QS powder could not increase IgG titers. Neither QS nor CDM adjuvant could significantly increase the lavage anti-TT IgA titers. 
